# Comparative on the effectiveness and safety of different carotid endarterectomy techniques: a single-center Retrospective Study

**DOI:** 10.1186/s13019-024-02838-0

**Published:** 2024-06-20

**Authors:** Sensen Wu, Hui Wang, Julong Guo, Fan Zhang, Dikang Pan, Yachan Ning, Yongquan Gu, Lianrui Guo

**Affiliations:** Department of Vascular Surgery, Xuanwu Hospital, Capital Medical University, Beijing, China

**Keywords:** Carotid artery stenosis, Carotid endarterectomy, Stroke

## Abstract

**Background:**

Carotid endarterectomy (CEA) is a surgical procedure that can reduce the risk of stroke in patients with carotid artery stenosis. However, controversy still exists regarding the optimal surgical technique for CEA.

**Objective:**

To compare the safety and effectiveness of different techniques.

**Methods:**

Data on baseline characteristics as well as perioperative and postoperative complications from patients who underwent CEA at the Department of Vascular Surgery, Xuanwu Hospital, Capital Medical University, were retrospectively collected and analyzed.

**Results:**

A total of 262 CEA patients included in study, with a total of 265 CEA operations. The mean age of 69.95 ± 7.29 (range, 44–89) years. 65 (24.5%) patients underwent cCEA, 94 (35.5%) underwent pCEA, and 106 (40.0%) underwent eCEA. The use of shunt (1.9%) and the mean operation time were lower in eCEA group (*P* < 0.05). eCEA was also associated with a lower incidence of postoperative hypotension, whereas pCEA was associated with a lower incidence of postoperative hypertension (*P* < 0.05). There was no significant difference in clinical baseline characteristics, occurrence of perioperative complications, and survival whether restenosis-free, asymptomatic or overall.

**Conclusions:**

This study found that all three surgical methods are equally safe for the treatment of carotid artery stenosis and are effective in preventing stroke.

## Introduction

With the acceleration of population aging and urbanization, along with the evident increase in stroke risk factors, the incidence of stroke overall is on the rise. One of the primary causes of ischemic stroke is carotid artery stenosis, with approximately 90% of cases attributed to atherosclerosis, leading to transient ischemic attacks (TIA) or stroke [1, 2]. Surveys indicate that the prevalence rate of severe atherosclerotic carotid stenosis (stenosis degree ≥ 70%) in the general population is estimated to range from 0.1 to 3%, with higher rates observed in men, patients with coronary heart disease, and the elderly (aged ≥ 65 years) [3].

In 1960s, Debakey [4] completed the first case of carotid endarterectomy (CEA) to prevent stroke and achieved satisfactory results during the 19-year follow-up process. Building on this landmark achievement, surgeons have continued to refine the technique of carotid endarterectomy [5, 6]. Currently, carotid endarterectomy is considered one of the most effective methods for preventing stroke in patients with carotid stenosis. Despite the emergence of new brain protection devices, equipment, and stents that have brought carotid artery stenting (CAS) to a comparable level in terms of perioperative complications and long-term patency [7]. CEA remains the preferred treatment for carotid stenosis according to current guidelines [8, 9]. Among these interventions, carotid artery revascularization plays a pivotal role in preventing stroke and improving patient outcomes. However, the choice between surgical techniques remains a subject of ongoing debate and clinical decision-making.

The most commonly used surgical methods for CEA as reported in the literature include conventional carotid endarterectomy (cCEA), patch carotid endarterectomy (pCEA), and eversion carotid endarterectomy (eCEA). However, there is still no definitive evidence regarding the choice between these three surgical methods. The 2023 guideline from the European Society of Vascular Surgery (ESVS) recommends patch and eversion CEA as the preferred choices, with the surgeon making the decision between eversion or patch CEA [8]. However, the majority of studies included in this proposal are based on data from 10 or even 20 years ago. Given the significant advancements in surgical techniques, care and best medicine treatment, the data from these trails may no longer be applicable to the current medical environment. Therefore, the purpose of this study is to compare the safety and effectiveness of three different CEA techniques in our unit and to generate our own data regarding the advantages and disadvantages of these three procedures.

## Materials and methods

### Patients

This study retrospectively analyzes all patients who underwent CEA for carotid artery stenosis at the Department of Vascular Surgery, Xuanwu Hospital, Capital Medical University from 2018 to 2022. All included patients were divided into cCEA group, pCEA group and eCEA group. All patients had signed an informed consent form before surgery. The inclusion criteria of this study were: 1). Patients were diagnosed as carotid artery stenosis caused by atherosclerosis and performed carotid endarterectomy; 2). Age ≥ 45 years old, complete clinical data; 3). Symptomatic patients with carotid artery stenosis degree greater than 50%, or asymptomatic patients with stenosis degree greater than 70% (confirmed by carotid artery ultrasound, CTA or DSA.)

The primary outcomes of the study were rates of postoperative new ipsilateral stroke and long-term patency. The secondary outcomes were rates of overall survival, all-cause death, long-term recurrent stroke and occurrence of postoperative complications, such as hemorrhage, neck hematoma, cranial nerve injury, and hyperperfusion syndrome.

## Data collection

Baseline characteristics, including age, gender, neurological symptoms, degree of carotid artery stenosis, smoking and alcohol consumption history, and previous medical history (such as coronary artery disease, diabetes, hypertension), were collected. Intraoperative data were also collected, including surgery time, intraoperative blood loss, and shunt use. Additionally, blood pressure, heart rate and major complications during hospitalization were recorded.

Carotid artery ultrasound and transcranial Doppler ultrasound (TCD) were performed in all patients before surgery to determine the degree of stenosis and contralateral carotid artery patency. The degree of carotid artery stenosis was according to the criteria of the North American Symptomatic Endarterectomy Trial (NASCET) [10].

### Surgical techniques

All patients received a single antiplatelet medication (either aspirin 100 mg or clopidogrel 75 mg) combined with statins for at least three days prior to surgery. The surgery was performed under general anesthesia and tracheal intubation, and a TCD monitoring system was installed as routine to monitor real-time changes in intracranial blood flow. After systemic heparinization, the internal carotid artery, common carotid artery, and external carotid artery were separated and clamped with vascular blocking forceps. The blood flow velocity of the ipsilateral middle cerebral artery in the TCD system was then monitored after administering a pressure boosting drug. An intraoperative shunt was used if the blood flow velocity of the middle cerebral artery decreased by more than 50% after blocking.

#### cCEA

The vessel wall is cut along the common carotid artery and the internal carotid artery. The subintimal atherosclerotic plaque is completely removed. After thoroughly irrigating the vascular lumen, the lateral wall is sutured continuously in situ.

#### pCEA

Based on cCEA, instead of directly suturing the vascular wall, artificial patches are used for repair and suturing onto the vascular wall.

#### eCEA

At the bifurcation of the common carotid artery, the internal carotid artery is cut off in an arc. The outer membrane of the internal carotid artery is clamped, and a sleeve-style dissection of the atherosclerotic plaques is performed up to the distal end. Additionally, the proximal end at the common carotid artery and the distal end at the point of thinning of the internal carotid artery plaques are removed. Simultaneously, the inner membrane is peeled off from the external carotid artery. The vascular lumen is thoroughly rinsed before performing an end-to-end anastomosis between the internal carotid artery and the common carotid artery.

After surgical anastomosis was completed, the common carotid artery and external carotid artery were sequentially opened, followed by the internal carotid artery. After confirming good TCD monitoring blood flow signal and thorough control of incisional bleeding, a drainage tube was placed, and the surgical incision was closed layer by layer.

Postoperatively, patients were regularly monitored in the intensive care unit for vital signs and neurological function status. Generally, systolic blood pressure was maintained between 110-140mmHg or less than 20% of the baseline, and blood pressure was kept stable. Dual antiplatelet therapy (aspirin 100 mg + clopidogrel 75 mg) was initiated on the second day after surgery.

### Follow up

If there are no contraindications, all patients undergoing CEA will continue to receive combination therapy with dual antiplatelet therapy (aspirin 100 mg + clopidogrel 75 mg) along with statins. Antiplatelet therapy could be changed at physician’s discretion to a single drug combined with statins during the outpatient review three months later.

Follow-up will be conducted for all enrolled patients, primarily through telephone and outpatient visits. The follow-up intervals will be at 3 months, 6 months, 12 months, and thereafter yearly. The primary outcome measures will include the occurrence of new strokes, restenosis (defined as a stenosis degree ≥ 50%), death, and the causes of death.

### Statistical analysis

All statistical analyses were conducted using SPSS 25.0 software. Normality testing was performed on econometric data using mean ± standard deviation (x ± s) to represent a normal distribution. One-way ANOVA was used to compare data that followed a normal distribution. For comparisons between three groups, the counting data was expressed as numerical percentages and chi-square tests were used. Fisher’s exact test was used when the actual sample size was less than 5. Bonferroni’s correction was used to compare differences between groups when *P* < 0.05. The Kaplan Meier method was used to analyze the survival of restenosis-free, asymptomatic or overall. A *P* < 0.05 indicated that the difference was statistically significant.

## Results

### Baseline data

The study involved 262 patients who underwent CEA surgery at the department of vascular surgery at Xuanwu Hospital, Capital Medical University from January 2018 to January 2022, with a total of 265 CEA surgeries performed. Three patients underwent staged bilateral surgery. The majority of patients were male, accounting for 84.9% of the total proportion, with an age range of 44 to 89 years and an average age of 69.95 ± 7.29 years. Based on the surgical method, 65 patients (24.5%) underwent cCEA, 94 patients (35.5%) underwent pCEA, and 106 patients (40.0%) underwent eCEA.

Table 1 presents an overview of the baseline data for the three study groups. As indicated in Table 1, there were no statistically significant differences among the three groups in terms of gender, age, side, neurological symptoms, hypertension, diabetes, coronary heart disease, and other underlying conditions, as well as smoking and drinking history. Furthermore, there was no statistically significant difference in the degree of bilateral carotid artery stenosis or the presence of ulcerative plaques.

**Table 1:** Baseline characteristics

	cCEA	pCEA	eCEA	*P*
N (%)	65(24.5%)	94(35.5%)	106(40.0%)	-
Male	57(87.7%)	78(83%)	90(84.9%)	0.72
Age, year	64.52 ± 6.68	64.12 ± 7.52	63.42 ± 7.48	0.59
Left side	38(58.5%)	44(46.8%)	47(44.3%)	0.18
Hypertension	43(66.2%)	56(59.6%)	66(62.3%)	0.70
Diabetes	20(30.8%)	36(38.3%)	36(34%)	0.61
Coronary artery disease	13(20%)	19(20.2%)	19(17.9%)	0.91
Smoking	31(47.7%)	41(43.6%)	58(54.7%)	0.28
Drinking	19(29.2%)	28(29.8%)	36(34%)	0.75
Asymptomatic	39(60%)	65(69.1%)	70(66%)	0.49
Ulcerative plaque	13(20%)	19(20.2%)	19(17.9%)	0.91
Degree of stenosis				
50–69%	14(21.5%)	22(23.4%)	26(24.5%)	0.90
≥ 70%	51(78.5%)	72(76.6%)	80(75.5%)	0.90
Contralateral stenosis ≥ 50%	12(18.5%)	24(25.5%)	24(22.6%)	0.58

### Perioperative data

All patients successfully completed the surgery without any perioperative deaths. Table 2. presents the perioperative clinical data of three groups of patients. There are statistical differences among the three groups in terms of surgical time, use of shunt, and postoperative blood pressure (*P* < 0.05). Further analysis revealed that the average surgical time for eCEA was 127.95 ± 37.18 min, which was significantly lower than the other two groups (*P* < 0.01). The use of shunt in eCEA group was significantly lower than that of the other two groups (*P* < 0.01). Postoperative blood pressure monitoring showed that eCEA was associated with a lower incidence of postoperative hypotension (0.9%), while pCEA was associated with a lower incidence of postoperative hypertension (31.9%) (*P* < 0.01). There was no significant difference among the three groups of patients in postoperative hospitalization time, intraoperative bleeding and emergency surgery.

**Table 2:** Perioperative data

	cCEA(*n* = 65)	pCEA(*n* = 94)	eCEA(*n* = 106)	*P*
Surgery time (min)	156.95 ± 54.89	157.73 ± 41.8	127.95 ± 37.18	<0.01
Shunt	22(33.8%)	44(46.8%)	2(1.9%)	<0.01
Intraoperative bleeding (mL)	42.85 ± 35.55	55 ± 83.82	34.62 ± 51.92	0.07
Postoperative hypertension	31(47.7%)	30(31.9%)	56(52.8%)	<0.01
Postoperative hypotension	9(13.8%)	9(9.6%)	1(0.9%)	<0.01
Emergency operation	1(1.5%)	1(1.1%)	4(3.8%)	0.58
Length of postoperative stay (d)	6.45 ± 1.87	6.50 ± 1.99	7.68 ± 6.81	0.11

### Complications data

The incidence of perioperative complications was calculated, as shown in Table 3. Overall, there was no statistically significant difference in the incidence of perioperative complications among the three groups. The most common complication after CEA was cranial nerve injury, with an overall incidence rate of 6.8%, followed by neck hematoma, with an incidence rate of 4.15%. Among them, six cases required emergency surgical. The incidence of neck hematoma was higher in the eCEA group (6.6%) compared to the other groups, but this difference was not statistically significant. There were 6 cases of perioperative stroke (with neurological symptoms), with an overall incidence rate of 2.7%. There was 1 case of postoperative acute thrombosis in the eCEA group, but there were no obvious neurological symptoms. There were 2 cases of acute thrombosis in the pCEA group, with 1 case undergoing emergency interventional thrombectomy due to neurological symptoms.

**Table 3:** Complications data

	cCEA(*n* = 65)	pCEA(*n* = 94)	eCEA(*n* = 106)	*P*
Death	0	0	0	-
Cranial nerve injury	5(7.7%)	6(6.4%)	7(6.6%)	0.91
Hyperperfusion syndrome	1(1.5%)	3(3.2%)	2(1.9%)	0.77
Perioperative stroke	1(1.5%)	3(3.2%)	2(1.9%)	0.77
Neck hematoma	2(3.1%)	2(2.1%)	7(6.6%)	0.33
Acute thrombosis	0(0%)	2(2.1%)	1(0.9%)	0.62
Over-all	9(13.8%)	16(17.0%)	19(18.3%)	0.79
Residual stenosis	1(1.5%)	3(3.2%)	1(0.9%)	0.54

### Follow up

Follow-up was conducted on all included patients, with a median follow-up time of 26 months and an average follow-up time of 28 ± 14 months. During the follow-up period, a total of 14 cases (5.3%) developed restenosis (stenosis degree > 50%). Eleven patients (4.2%) developed neurological symptoms. Six cases (2.3%) died during the follow-up period. The follow-up results are shown in Table 4.

**Fig. 1:**
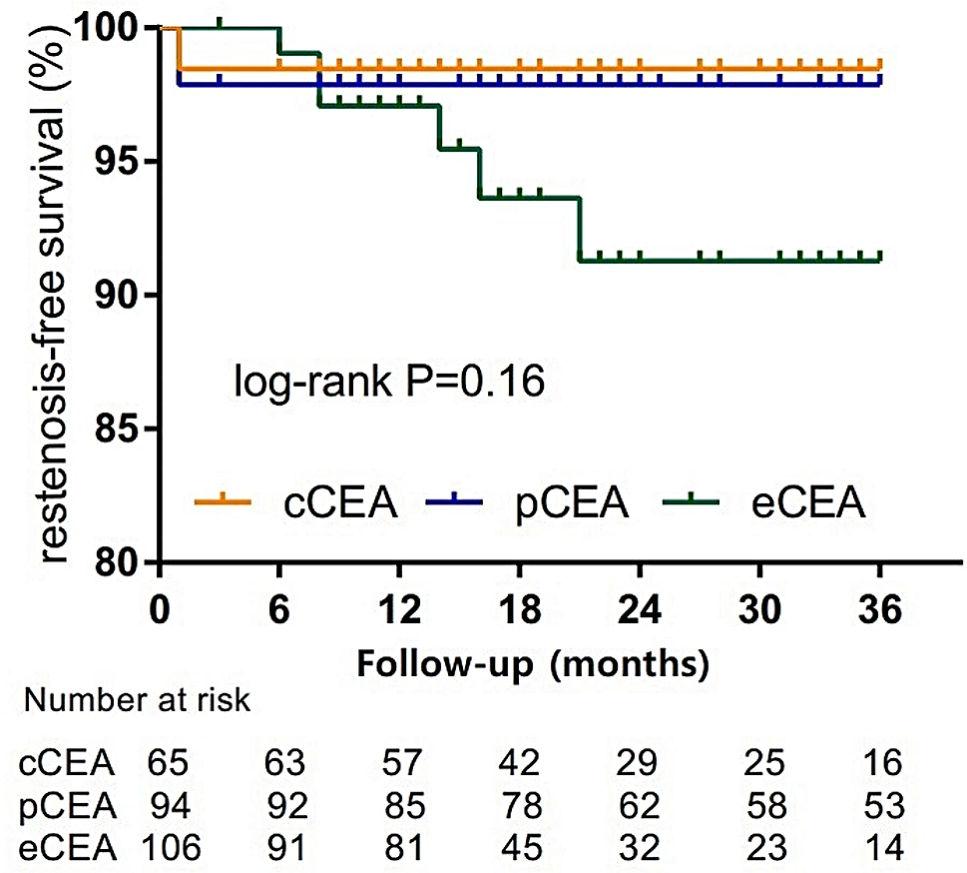
The three-year restenosis-free survival rate

**Fig. 2:**
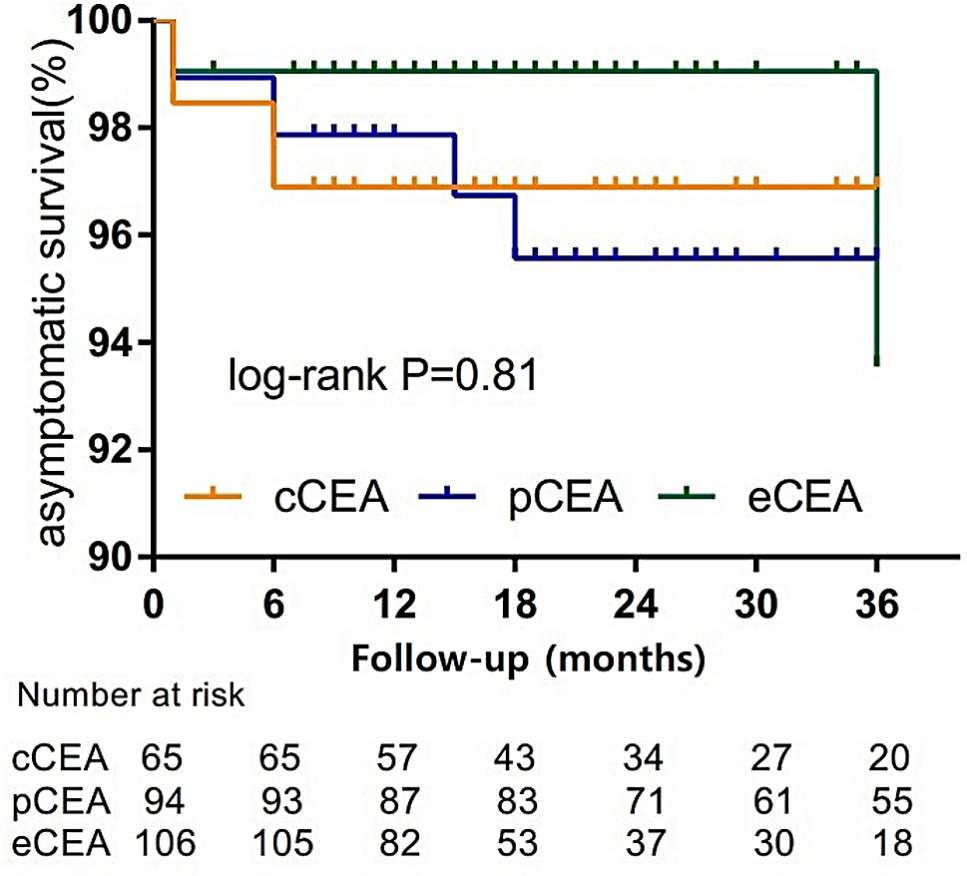
The 3-year asymptomatic survival rate

**Fig. 3:**
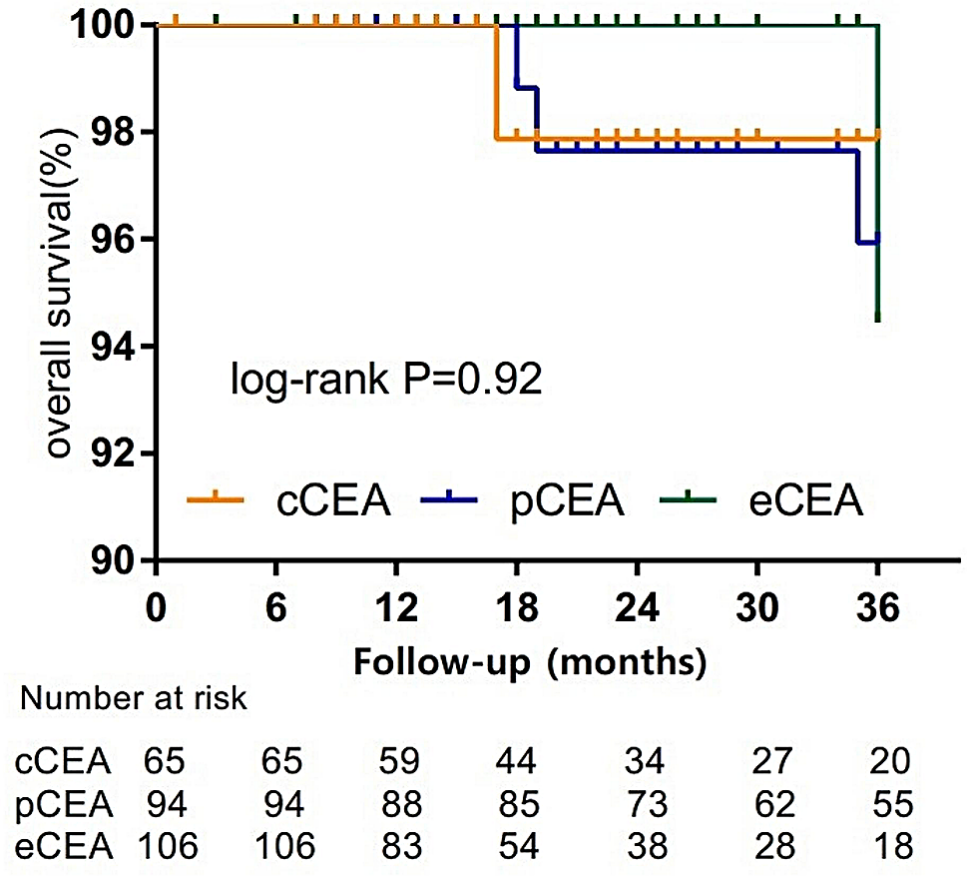
The three-year overall survival rate of the three groups of patients

According to the patient’s follow-up data, a survival analysis curve was plotted. Figure 1 displays the three-year restenosis-free survival rate. Figure 2 illustrates the 3-year asymptomatic survival rate. Figure 3 presents the three-year overall survival rate of the three groups of patients. The log-rank test reveals no statistical difference among the three groups.

**Table 4:** Follow-up data

	cCEA(*n* = 65)	pCEA(*n* = 94)	eCEA(*n* = 106)	*P*
Follow-up duration (m)	26 ± 13	35 ± 14	21 ± 11	-
Restenosis	2(3.1%)	4(4.3%)	8(7.5%)	0.4
Stroke	3(4.6%)	6(6.4%)	2(1.9%)	0.24
All-cause mortality	1(1.5%)	4(4.3%)	1(0.9%)	0.29

## Discussion

In 1953, DeBakey [4, 5]et.al completed the first case of CEA to prevent stroke, and achieved positive outcomes during the follow-up for over ten years. Since then, CEA has gradually gained acceptance as a preventative method for ischemic stroke. Initially, the effectiveness and safety of CEA in stroke prevention were unclear, leading to some doubts. However, the publication of the results from two large-scale randomized controlled trials, NASCET [11] and ECST [12], in the 1990s officially confirmed CEA as the " preferred method " for treating carotid artery stenosis. Over the course of 60 years, CEA techniques have undergone continuous innovation and improvement, resulting in variations such as pCEA and eCEA. The eversion technique was first proposed by DeBakey [6] in 1959 and later popularized by Etheredge [13], Subsequently, Kasprzak、Raithel [14]and Vanmaele [15]et al. made further improvements to eversion CEA, extending its utilization to the present day. Additionally, the use of different materials, transverse incisions to minimize scarring, and the identification of the optimal surgical approach to reduce nerve damage are among the various innovations that have consistently enhanced the clinical effectiveness of CEA [16–18].

CEA as one of the effective methods for stroke prevention, has always been a concern for its perioperative safety. Randomized controlled trials have shown that 19-24% of perioperative strokes and deaths occur after the eighth day following the operation [19]. Therefore, stroke, death, and stroke/death rates within 30 days after CEA operation are important indicators for evaluating a unit’s ability to perform CEA procedures. With advancements in surgical techniques and best medical treatments, there is sufficient evidence to demonstrate a gradual decrease in the incidence of stroke and death within 30 days after CEA. A study analyzed data from 6 trials and 47 community registries conducted between 1983 and 2013, revealed a significant reduction in the incidence of postoperative stroke or death after CEA surgeries performed from 1991 to 2010. The study concluded that CEA is now safer than ever, with a stroke or death risk of 1.2% and a death risk of 0.4% [20]. Therefore, some experts [8] suggest that the current rate of 6% should be further reduced. They propose that the 30-day stroke death rate for symptomatic patients should be less than 4% and for asymptomatic patients it should be less than 2%. These suggestions aim to improve the surgical outcomes by lowering the threshold.

Many studies have compared the perioperative safety of pCEA with cCEA. The majority of these studies demonstrate that pCEA can effectively reduce the risk of ipsilateral stroke and arterial occlusion during the perioperative period [21, 22]. EVEREST [23] is a randomized multicenter trial comparing the effects of three surgical methods. The results indicate that the incidence of major stroke and death within 30 days in the perioperative period is similar among the three surgical methods. Additionally, there is no significant difference in the incidence of all strokes in the perioperative period. A recent meta-analysis [24] comparing eCEA with cCEA demonstrated that eCEA does not have an impact on reducing stroke, death/stroke, or death/stroke myocardial infarction within 30 days in five randomized controlled trials. However, in 20 observational studies, eCEA was associated with a significant reduction in 30-day mortality, stroke, and death/stroke/myocardial infarction. When comparing eCEA with pCEA, it was found that there was no significant difference in the main outcome measures between the two methods. Huizing [22]et al found that the risk of 30-day stroke in the cCEA group was higher (OR, 1.9; 95% CI 1.2–2.9). However, when excluding non-randomized studies, the difference was no longer statistically significant (OR, 1.9; 95% CI, 0.8–3.9). In our research, we found that the overall incidence of perioperative stroke and death after CEA was 2.7%. There was no statistical difference in the incidence rates of perioperative stroke and death among the three groups (*P* = 0.768). Our research results were similar to those of the EVEREST [23], which also showed that all three surgical methods were associated with perioperative stroke and death.

Neck hematoma is a serious complication that can occur after CEA. In severe cases, it can lead to compression of the trachea, resulting in upper airway obstruction and dyspnea. The use of perioperative antiplatelet drugs and systemic heparinization during the operation has made neck hematoma increasingly common as a complication after CEA. A study [25] had shown that patients with hematoma have significantly increased transfusion, mortality, perioperative stroke and myocardial infarction rates, as well as prolonged hospitalization times. Additionally, a study [26] on predictive factors for unplanned readmission within 30 days after CEA found that bleeding and neck hematoma increase the risk of readmission within this timeframe (HR: 3.1; 95% CI: 1.4–6.9, *P* = 0.003). Paraskevas [24] found that there was no significant statistical difference in the incidence of hematoma between eCEA and cCEA (2.7% vs. 2.04%, OR: 1.2), but eCEA was superior to pCEA (3.04% vs. 3.62%, OR: 0.53, *P* = 0.03).

Cerebral hyperperfusion syndrome (CHS) is a postoperative complication of carotid endarterectomy, although the incidence of this syndrome is low, ranging from 1 to 3%, it can be fatal, with reported mortality rates as high as 50%. Grace [27] conducted a statistical analysis using the Vascular Quality Initiative (VQI) database to examine patients undergoing CEA from 2003 to 2015. They identified that the overall incidence rate of CHS was 0.18%, with a mortality rate of 38.2%. Their analysis further revealed strong associations between CHS and factors such as female, recent severe stroke, coronary heart disease, contralateral severe carotid stenosis and postoperative blood pressure instability. Another study [28] supported that strict intraoperative blood pressure control may be effective strategies to decrease the occurrence of CHS and its associated complications.

Cranial nerve injuries receive less attention than other serious complications, but bilateral injuries can be fatal. It has been reported that the vagus nerve is the most frequently injured cranial nerve during CEA, with an incidence rate of 3.99%. This is followed by the hypoglossal nerve (3.79%) and mandibular marginal nerve (1.58%). Injuries to the glossopharyngeal nerve and spinal cord accessory nerve are quite rare, with rates of 0.22% and 0.21% respectively [29]. Furthermore, long-term follow-up results of patients with cranial nerve injuries showed that most cases have benign outcomes, with high rates of symptom regression and extremely low incidence of persistent clinically significant symptoms [30].

Our study found that cranial nerve injury is the most common complication, with an incidence rate of 6.8%. During the follow-up period, most patients experienced relief of their symptoms within approximately 1–6 months after the operation, and no cases of permanent injury were identified. The next most frequent complication was neck hematoma, with an incidence rate of 4.15%. Among these cases, 6 (54.5%) patients required emergency surgical. Our research data are different from those of Paraskevas [24], whose research shows that the incidence of hematoma in eCEA is lower than that in pCEA, in our study, there is no significant statistical difference in the incidence of hematoma among the three groups. The incidence of CHS in our unit was 2.26%, among which 4 cases showed ipsilateral headache and 1 case showed epileptic seizure, all of which occurred within 48 h after operation. Patients were treated with symptomatic treatment and no complications such as intracranial hemorrhage and neurological dysfunction occurred.

The earliest randomized controlled studies [31, 32] all indicated that CEA can significantly reduce the incidence of stroke and disabling stroke in asymptomatic patients within 5 years. Halliday [33] confirmed the long-term effectiveness of CEA in asymptomatic patients. A recent study [34] found that there is a correlation between the degree of carotid artery stenosis and the risk of ipsilateral stroke. The study showed that patients with carotid artery stenosis of 70-99% have a higher risk of stroke compared to those with stenosis of 50-70% (OR 2.1, *P* < 0.01). As a result, it is suggested that patients with severe stenosis (> 70%) may benefit more from undergoing CEA surgery.

A recent study [35] showed that the cumulative restenosis risk of the eCEA treatment group was significantly reduced for 4 years (3.6% vs. 9.2%, *p* = 0.01). However, there was no significant difference in the cumulative restenosis risk between the pCEA and eCEA (1.5% vs. 2.8%). Paraskevas [24] had shown that in both randomized controlled studies and observational studies, eCEA is associated with a reduction in late restenosis (OR = 0.45; *P* = 0.004). Similarly, there were no statistically significant differences in the long-term effectiveness of eCEA compared to pCEA or cCEA. Huizing [22] found that the long-term restenosis rate in the cCEA group was higher compared to eCEA (OR 2.2, 95% CI 1.4–3.4). Furthermore, some studies have shown that eCEA reduces ischemia (clamping time) and operation time, and during follow-up, the incidence of restenosis and anastomotic pseudoaneurysm is low. As a result, eCEA is considered to be the preferred choice for carotid artery stenosis.

Our study is similar to that of Cheng [36] in terms of the results obtained from the follow-up study of patients with carotid stenosis after 1 year and 5 years. Specifically, we found that there is no significant difference in the postoperative stroke risk among the three methods. Our study had a median follow-up time of 26 months, and in order to minimize error, we generated a three-year restenosis-free survival curve. The three-year restenosis-free survival rates for the three patient groups were 98.5%, 97.8%, and 91.3%, respectively. Unlike most previous studies, we did not find a significant difference in long-term patency rate between three groups in our study.

Unstable blood pressure following CEA increases the risk of neurological and cardiovascular complications. Unstable blood pressure post-endarterectomy is considered a risk factor for hyperperfusion syndrome [27]. The choice of different surgical methods and intraoperative procedures may impact postoperative blood pressure. Previous studies have found that eCEA is associated with postoperative blood pressure increase, while cCEA is associated with postoperative hypotension [37, 38]. However, long-term follow-up studies have not shown a significant impact of surgical methods on blood pressure control [39]. In our study, similar results were obtained. The incidence of perioperative hypotension in eCEA group was lower (0.9%) (*P* < 0.01), but the incidence of postoperative hypertension was higher (52.8%), requiring additional antihypertensive medication for blood pressure control. Notably, pCEA had the lowest incidence of postoperative hypertension (31.9%) (*P* < 0.01). Hence, we recommend closely monitoring blood pressure, especially when performing eversion CEA.

In this study, we conducted an in-depth comparison of clinical outcomes of different surgical techniques. We found that although all methods have significant therapeutic effects, there are certain differences in the incidence of postoperative complications and long-term prognosis. These differences can be attributed to the complexity of the technology itself, differences in intraoperative control variables, or individual patient factors. Comprehensiveness of preoperative evaluation, accuracy of intraoperative control, and implementation of postoperative rehabilitation plan. We further point out that the optimization of these factors can significantly reduce the risk of postoperative complications and improve the recovery speed of patients. Although our research provides some valuable insights, further research is needed to determine the optimal surgical techniques and perioperative management strategies. Especially in multicenter, large-scale studies, we can better validate our findings and provide a stronger evidence base.

### Limitations

This study is a single-center retrospective study. First, patients’ data are entered in the medical record system instead of first-hand information collection. At the same time, we may be biased in the choice of surgical methods, such as conventional or patch repair when shunt is needed, and when the diameter of internal carotid artery is small, it is more inclined to patch during operation. Although the overall sample size of our study is considerable, there are few positive data due to the low incidence of postoperative stroke, restenosis and perioperative complications, and there may be some errors in statistical analysis. Although there are some limitations, our research still shows that under the current situation of the best medical treatment, the occurrence of perioperative adverse events in the three groups of operations has tended to be similar, and it is necessary to reconsider the choice of the best surgical method in the contemporary environment, and a large-scale prospective multicenter study is still needed to verify it in the later stage.

## Conclusion

This single-center retrospective study showed that all three surgical methods are both safe and effective for treating carotid artery stenosis. No significant difference was observed in the three-year asymptomatic survival rate and the three-year no-stenosis survival rate. The findings of this study imply that the eversion CEA procedure has a shorter operation time and a lower incidence of postoperative hypotension. On the other hand, patch CEA has the advantage of a low incidence of postoperative hypertension.

## Data Availability

No datasets were generated or analysed during the current study.
